# Systematic review of the efficacy and safety of lenvatinib vs. sorafenib as first-line treatments for hepatocellular carcinoma

**DOI:** 10.1016/j.clinsp.2026.100869

**Published:** 2026-02-19

**Authors:** Wentao Bo, Zhenguo Wang, Xiuhua Dong, Jing Zou

**Affiliations:** a Department of Hepatopancreatobiliary Surgery, Sichuan Clinical Research Center for Cancer, Sichuan Cancer Hospital & Institute, Affiliated Cancer Hospital of University of Electronic Science and Technology of China, Chengdu, China; b Department of Stomatology, The 1st Affiliated Hospital of Chengdu Medical College, Chengdu, China

**Keywords:** Lenvatinib, Sorafenib, Hepatocellular carcinoma, PFS, Adverse event

## Abstract

**Objective:**

This study aimed to explore the efficacy and safety of lenvatinib and sorafenib as first-line treatments for hepatocellular carcinoma.

**Method:**

The authors searched PubMed, Web of Science, Cochrane, CNKI, Wanfang, and other databases and compared the clinical efficacy of lenvatinib and sorafenib as first-line treatments for hepatocellular carcinoma. Two participants screened the literature, collated the data, and evaluated the literature according to the inclusion and exclusion criteria. RevMan 5.4 software was used for meta-analysis of the included studies.

**Results:**

Ten studies were included in the meta-analysis. The results of the meta-analysis showed that, in terms of efficacy, compared with sorafenib, lenvatinib prolongs PFS in patients with hepatocellular carcinoma (HR = 85.50, 95 % CI: 38.53‒189.73, *p* < 0.00001) and OS (HR = 36.73, 95 % CI: 20.28–66.52, *p* < 0.00001), and the differences were statistically significant. In terms of safety, the risk of toxicities in the lenvatinib group at any level of gastrointestinal toxicities, metabolism/nutrition toxicities, hematological toxicities, Renal/Urinary, Vascular toxicities, and endocrine toxicities was significantly higher in the lenvatinib group than in the sorafenib group. The risks of metabolism/nutrition toxicities, renal/urinary toxicities, and vascular toxicities above grade III were significantly higher than those in the sorafenib group. The Skin/Subcutaneous Tissue toxicities of any grade and above were significantly lower than those in the sorafenib group.

**Conclusions:**

As a first-line treatment for hepatocellular carcinoma, lenvatinib can prolong PFS and OS and improve the clinical benefit rate and quality of life of patients. The increased risk of specific adverse events with lenvatinib requires diligent clinical oversight.

## Highlights

• Lenvatinib was associated with a higher incidence of ascites and bilirubin elevation, highlighting the need for vigilant liver function monitoring.
• Lenvatinib’s inhibition of VEGFR, FGFR, and PDGFR pathways contributes to its superior anti-angiogenic and anti-proliferative effects.
• Preclinical evidence suggests lenvatinib may synergize with immunotherapy by modifying the tumor microenvironment, offering novel therapeutic avenues.
• Economic analyses indicate lenvatinib is more cost-effective than sorafenib, supporting its adoption in resource-limited settings.

## Graphical abstract

The results demonstrate that Lenvatinib significantly prolongs both Progression-Free Survival (PFS) and Overall Survival (OS) when compared to Sorafenib. The safety profile indicates that the Lenvatinib group had a higher risk of toxicities, including nausea, diarrhea, decreased appetite, and hypertension.

**Figure:**
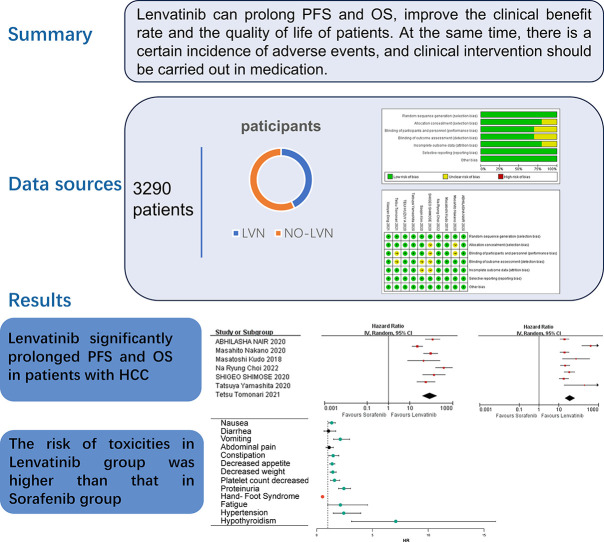

## Introduction

Primary liver cancer is a globally prevalent malignant tumor of the digestive system. In 2020, the world witnessed 905,677 new cases and 830,180 deaths due to this disease. Among all cancer types, primary liver cancer ranks sixth in incidence and fourth in mortality worldwide. Notably, China accounts for approximately 45 % of these new cases, with 410,038 patients.^1^ These cancers can be categorized into Hepatocellular Carcinoma (HCC), intrahepatic cholangiocarcinoma, and other rare types.^2^ Major risk factors for HCC include infection with Hepatitis B Virus (HBV) or Hepatitis C Virus (HCV), consumption of aflatoxin-contaminated food, excessive alcohol consumption, obesity, type 2 diabetes, and smoking.^3^ Currently, the primary treatment options for HCC include surgical resection, radiofrequency ablation, and others. However, owing to its insidious onset, most patients are diagnosed at the middle or advanced stages. In the absence of effective treatment, the natural survival duration is merely 3‒4 months, posing a serious threat to human health. Systemic therapy remains a viable option to delay disease progression in patients ineligible for local treatments, such as surgery. Targeted therapy, characterized by targeting precision, high specificity, resistance to drug tolerance, remarkable efficacy, and minimal side effects, is a pivotal component of systemic therapy and has emerged as a focal point in HCC treatment research. These drugs specifically bind to identified carcinogenic sites in HCC, thereby exerting anticancer effects. Recently, with the unveiling of additional mechanisms underlying HCC occurrence and development, along with the identification of high-risk factors for liver cancer, continuous advancements in HCC diagnosis, treatment, prognosis, and molecular markers, and the progression of clinical trials for new drugs related to HCC molecular-targeted therapy, a multitude of HCC molecular-targeted drugs have been introduced into the market.

HCC-targeted therapeutic drugs exert their effects by targeting specific molecules or pathways involved in cancer cell growth and proliferation. Tyrosine Kinase Inhibitors (TKIs), such as sorafenib, regorafenib, and sunitinib, disrupt the cell signal transduction pathways essential for cancer cell growth and survival by inhibiting the activity of certain tyrosine kinases.^4^ Sorafenib, a multi-targeted oral antitumor agent, simultaneously inhibits RAF kinase, Vascular Endothelial Growth Factor Receptor-2 (VEGFR-2), Vascular Endothelial Growth Factor Receptor-3 (VEGFR-3), and Platelet-Derived Growth Factor Receptor-β (PDGFR-β), thereby directly or indirectly inhibiting tumor growth. Phase III clinical trials, including SHARP and Oriental studies, have demonstrated that sorafenib can significantly prolong the median survival time and delay disease progression.^5^ However, despite these benefits, the long-term survival rate remains low among patients with advanced liver cancer, and the recurrence rate after treatment remains high. Therefore, there is an urgent need to explore safer and more effective treatment options.

In recent years, advancements in HCC research have led to the introduction of several new drugs, including lenvatinib, donafinib, and carbotinib, which have been incorporated into HCC treatment guidelines as first- and second-line treatment options. The field of HCC molecular-targeted therapy is currently experiencing rapid development, with various drugs at different stages of research. Lenvatinib, a tyrosine kinase inhibitor approved for marketing in China in September 2018, targets vascular endothelial growth factor and platelet-derived growth factor receptors, blocking the expression of tumor vascular endothelial growth factor and inhibiting tumor angiogenesis, thereby exerting an antitumor effect. Additionally, lenvatinib can inhibit fibroblast growth factor receptor and rearrange it during transfection, thereby inhibiting tumor angiogenesis and proliferation.^6^ This dual mechanism of action can further modify the immunosuppressive tumor microenvironment and enhance antitumor immune responses.

A controlled trial conducted by Kudo et al.^7^ revealed that the Median Overall Survival (mOS), median time to progression (mTTP), and Objective Response Rate (ORR) of HCC patients treated with lenvatinib were 13.6-months, 8.9-months, and 24 %, respectively, surpassing those of the control group treated with sorafenib (12.3-months, 3.7-months, and 9 %). Choi et al.^8^ found that lenvatinib demonstrated superior clinical efficacy compared with sorafenib, particularly in hepatitis B-related HCC, with comparable incidence rates of adverse reactions (92.34 % vs. 93.09 %). Consequently, lenvatinib is the preferred option for patients in Asia and Africa, where hepatitis B-related HCC is prevalent. Various clinical trials have been conducted to assess the safety and efficacy of lenvatinib in the treatment of solid tumors. Given the relatively short clinical history of lenvatinib, this study conducted a meta-analysis of several controlled trials comparing the clinical efficacy of lenvatinib and sorafenib in the treatment of advanced primary liver cancer. The primary objective of this systematic review and meta-analysis extended beyond simply confirming the general efficacy advantage of lenvatinib over sorafenib in the first-line treatment of advanced hepatocellular carcinoma. A more critical aim was to address key clinical questions through quantitative synthesis. First, while pivotal Phase III trials like the REFLECT study established the non-inferiority and, in some endpoints, superiority of lenvatinib, the magnitude of this effect varied across studies due to population heterogeneity. By pooling available data, this study sought to provide a more precise and reliable consolidated Hazard Ratio (HR) for PFS and OS, thereby quantifying the treatment benefit to furnish the strongest possible evidence for clinical decision-making. Second, and of paramount importance, the choice of therapy necessitates a nuanced balance between efficacy and safety. This study, for the first time via meta-analysis, aimed to systematically characterize and compare the complete safety profiles of the two drugs across multiple organ systems and severity grades. Elucidating the specific differences in the type and incidence of adverse events is of vital importance for guiding individualized drug selection tailored to a patient's baseline comorbidities.

## Materials and methods

### Search strategy and study selection

PubMed, Web of Science, Cochrane, CNKI, and Wanfang databases. Were searched by computer, and the subject words + free words are used for document retrieval, and the references included in the literature are searched manually, so as to expand the retrieval scope and improve the recall rate. The retrieval time limit was from the establishment of the database until March 2024. In addition, the references included in the literature were traced back to the relevant literature. The search terms mainly included lenvatinib, E7080, lenvatinib mesylate, sorafenib, tumors, liver cancer, unresectable liver cancer, intermediate and advanced liver cancer, clinical trials, and randomized controlled trials.

The authors have specified the exclusion and inclusion criteria in advance. The literature included domestic and international clinical trials, RCTs, or retrospective studies of lenvatinib or sorafenib therapy in patients with advanced or unresectable liver cancer. Experimental group: lenvatinib alone; control group: sorafenib alone. The authors measured the outcome index of Hazard Ratio (HR) or Relative Risk (RR) and 95 % Confidence Interval (95 % CI) for PFS, OS, Clinical benefit (including Objective Response, Complete Response, Partial Response, Stable Disease, Progressive Disease), and adverse events (including hematological toxicities, gastrointestinal toxicities, metabolism/nutrition toxicities, Renal/Urinary toxicities, Skin/Subcutaneous Tissue toxicities, general toxicities, endocrine toxicities, and vascular toxicities).

Studies were excluded based on the following criteria: 1) Review, meta-analysis, case reports, summaries, editorials, letters to editors, etc.; 2) Republished literature; and 3) Non-Chinese and English literature; 4) The data were incomplete and could not be extracted; 5) Single-cohort, non-comparative study.

### Data extraction and quality assessment

Two evaluators independently screened the literature, extracted, and cross-checked the data. In the case of differences, consult a third party to help judge and contact the author as much as possible to supplement the lack of data. When selecting documents, first read the title and abstract and then read the full text after excluding obviously irrelevant documents to determine whether to include them in the end. The data extraction contents mainly include: 1) The first author/published year; 2) Basic characteristics of the subject; 3) Sample size; 4) Intervention plan; 5) Outcome index. For the original study with multiple subgroups, the data of the experimental and control groups related to this study were extracted.

The bias risk included in the study was evaluated by two evaluators using the bias risk quality evaluation tool for RCT in the Cochrane Manual.^9^

### Statistical analysis

RevMan 5.4 software was used for the meta-analysis. The count data were analyzed using Relative Risk (RR), and continuous data were analyzed using Mean Difference (MD) or Standardized Mean Difference (SMD). The combined effect and 95 % Confidence Interval (95 % CI) were simultaneously calculated at the same time. When conducting a meta-analysis, assessing the heterogeneity among study results is a crucial step. Heterogeneity refers to the differences in effect size estimates among different studies, which may stem from various factors such as differences in study design, sample characteristics, interventions, or measurement methods. The *I^2^* statistic is typically used to quantitatively assess the magnitude of this heterogeneity.

The *I^2^* statistic is an indicator used to quantify heterogeneity among the study results in a meta-analysis. It represents the proportion of the total variation attributable to between-study variation. A higher *I^2^* value indicates greater heterogeneity among the study results; conversely, a lower *I^2^* value suggests less heterogeneity.

After determining the magnitude of heterogeneity, the authors must select an appropriate meta-analysis model based on whether heterogeneity is present. If there is no statistical heterogeneity among the study results, that is, a lower *I^2^* value, the authors can use the fixed-effects model for meta-analysis. The fixed effects model assumes that all studies are randomly sampled from a common population, thus ignoring between-study variation and focusing only on within-study variation.

However, if there is statistical heterogeneity among the study results, that is, a higher *I^2^* value, the sources of heterogeneity need to be further analyzed. This includes examining differences in study design, sample characteristics, interventions, or measurement methods to determine which factors may contribute to inconsistencies among study results. After excluding the impact of obvious clinical heterogeneity, the authors adopted the random-effects model for the meta-analysis.

The random-effects model differs from the fixed-effects model in that it allows for between-study variation and assumes that each study is sampled from a different population. Therefore, in the random-effects model, the authors not only focused on within-study variation but also on between-study variation. This makes the random-effects model capable of providing more robust effect-size estimates and wider confidence intervals when heterogeneity is present.

By combining the *I^2^* statistic for quantitative assessment and selecting the appropriate meta-analysis model based on the presence of heterogeneity, the authors can more accurately assess differences among different study results and draw more reliable conclusions. Significant clinical heterogeneity was treated by subgroup analysis, sensitivity analysis, or descriptive analysis alone. According to the recommendation of the Cochrane Systematic Review Production Manual, when the number of included studies was ≥10, the publication bias test was performed using a funnel diagram.

## Results

### Basic characteristics and quality evaluation of literature

**Fig. 1:**
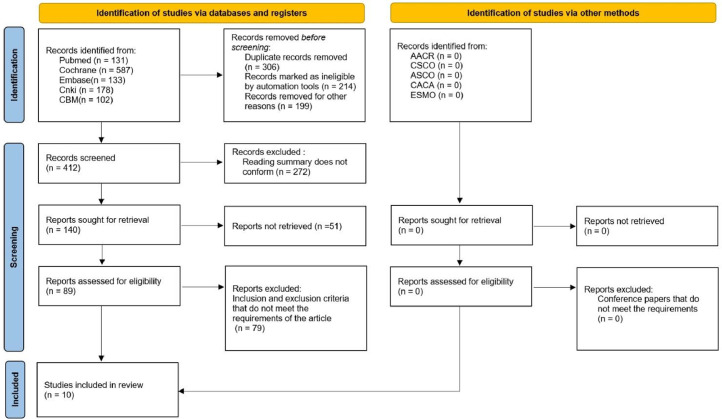
PRISMA Flow chart of article selection.

**Fig. 2:**
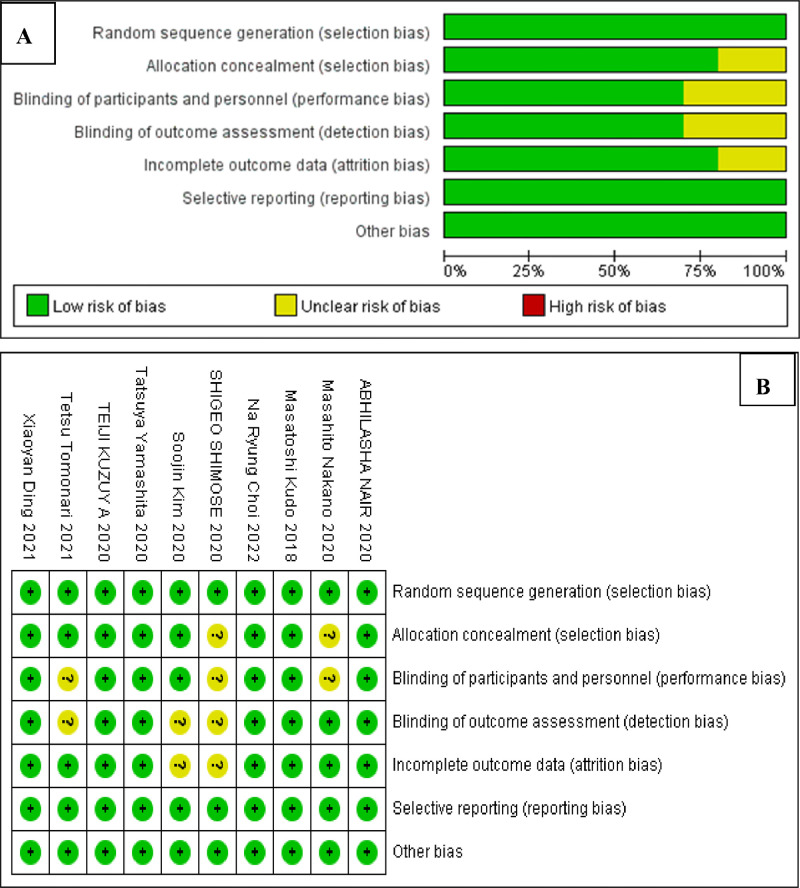
(A) The results of bias assessment. (B) Evaluation results of methodology quality of included studies.

According to the retrieval strategy and manual retrieval, 1131 studies were preliminarily obtained, and 10 studies were finally included after layer-by-layer screening,^8^^,^^9^^,^^11^, ^12^, ^13^, ^14^, ^15^, ^16^, ^17^, ^18^ with a total of 3290 patients, including 1413 cases in the lenvatinib group and 1877 cases in the non-lenvatinib group. The process and results of literature screening are presented in Fig. 1. The basic characteristics of the study are shown in Table 1, and the results of the bias risk assessment are shown in Fig. 2A and B.

**Table 1:** Basic characteristics of included studies.^10^, ^11^, ^12^, ^13^, ^14^, ^15^, ^16^, ^17^, ^18^, ^19^.

First Author	Year	Phase	Center	Treatment	Sample	Age	Gender (M/F)	ECOG (0/1)	Child-Pugh class (A/B)
Masatoshi Kudo[10]	2018	III	Multicentre	Lenvatinib	478	63 (20‒88)	405/73	304/174	475/3
				Sorafenib	476	62 (22‒88)	401/75	301/175	471/5
Xiaoyan Ding[11]	2021	III	Single-center	Lenvatinib	32	57 ± 11	25/7	24/8	22/10
				Sorafenib	32	56 ± 11	27/5	22/10	28/4
Na Ryung Choi[12]	2022	III	Single-center	Lenvatinib	44	58 (51.5‒64.8)	40/4	23/9	29/15
				Sorafenib	88	58 (52.3‒64.8)	80/8	48/29	63/25
Abhilasha Nair[13]	2020	III	Multicentre	Lenvatinib	478	63 (20‒88)	405/73	304/174	475/3
				Sorafenib	476	62 (22‒88)	401/75	301/175	471/5
Tatsuya Yamashita[14]	2020	III	Single-center	Lenvatinib	81	—	65/16	76/5	81/0
				Sorafenib	87	—	72/15	75/12	87/0
Teiji Kuzuy A[15]	2020	—	Single-center	Lenvatinib	13	70 (53‒92)	11/2	12/1	8/5
				Sorafenib	28	67 (35‒82)	21/7	10/18	13/15
Shigeo Shimose[16]	2020	—	—	Lenvatinib	45	75 (45‒89)	40/5	—	—
				Sorafenib	53	73 (54‒86)	46/7	—	—
Masahito Nakano[17]	2020	—	—	Lenvatinib	146	72.8 ± 9.6	125/21	—	134/12
				Sorafenib	524	70.9 ± 9.4	414/110	—	415/109
Soojin Kim[18]	2020	III	—	Lenvatinib	44	56 (51.0‒66.3)	39/5	41/3	36/8
				Sorafenib	61	64 (58.0‒70.5)	51/10	59/2	56/5
Tetsu Tomonari[19]	2021	—	—	Lenvatinib	52	70 (53‒88)	36/16	38/14	27/25
				Sorafenib	52	71 (43‒85)	35/17	37/15	27/25

Note: —, Not mentioned; M/F, Male/Female; ECOG, Eastern Cooperative Oncology Group.

### Outcome indicators

#### PFS

**Fig. 3:**
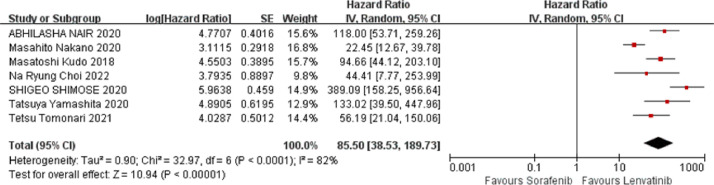
Meta-analysis results of PFS.

Seven studies reported PFS data, as shown in Fig. 3. The results of the heterogeneity analysis showed that there was heterogeneity among the studies (*I^2^* = 82 %, *p* < 0.0001), which was analyzed using a random effects model. Meta-analysis showed that compared with sorafenib, lenvatinib significantly prolonged PFS in patients with HCC (HR = 85.50, 95 % CI: 38.53‒189.73, *p* < 0.00001), and the difference was statistically significant.

#### OS

**Fig. 4:**
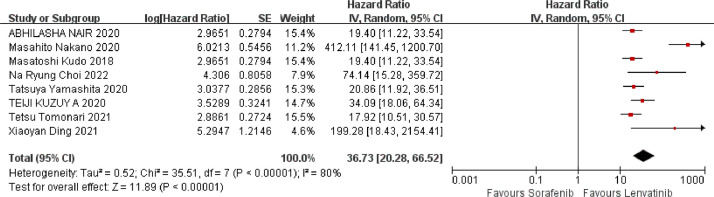
Meta-analysis results of OS.

Eight studies reported OS data, as shown in Fig. 4. The results of heterogeneity analysis showed that there was some heterogeneity among the studies (*I^2^* = 80 %, *p* < 0.00001), which was analyzed using a random-effects model. The meta-analysis showed that, compared with sorafenib, lenvatinib significantly prolonged OS in patients with HCC (HR = 36.73, 95 % CI: 20.28‒66.52, *p* < 0.00001), and the difference was statistically significant.

### Adverse event

#### Gastrointestinal toxicities

Gastrointestinal toxicities included nausea, diarrhea, vomiting, abdominal pain, and constipation. The results of the meta-analysis showed that, ① Compared with sorafenib, lenvatinib caused gastrointestinal toxicities at any level (RR = 1.29, 95 % CI: 1.04‒1.60, *p* = 0.02), the risk was relatively high, and the difference was statistically significant. Subgroup analysis showed that nausea (RR = 1.35, 95 % CI: 1.08‒1.68, *p* = 0.008), vomiting (RR = 2.12, 95 % CI: 1.52‒2.95, *p* < 0.0001), constipation (RR = 1.48, 95 % CI: 1.09‒1.99, *p* = 0.01), and the introduction of lenvatinib were significantly associated with a higher risk and has statistical difference. Among the gastrointestinal toxicities of grade III and above, lenvatinib-induced gastrointestinal toxicities (RR = 1.05, 95 %CI: 0.75‒1.47, *p* = 0.79) were similar to those in the sorafenib group, with no statistical significance. Subgroup analysis showed no statistical difference in nausea, diarrhea, vomiting, abdominal pain, and constipation above grade III between the two groups, as shown in Table 2, Table 3.

**Table 2:** Meta-analysis results of adverse event (Any grade).

Outcomes	Studies	Sample size	Heterogeneity		Effect model	Meta-analysis results	
			p	*I^2^*		95 % CI	p
**Gastrointestinal Toxicities**							
Nausea	4	2077	0.99	0	Fixed	1.35 [1.08, 1.68]	0.008
Diarrhea	9	3158	<0.00001	86	Random	1.06 [0.66, 1.71]	0.80
Vomiting	4	2077	0.98	0	Fixed	2.12 [1.52, 2.95]	<0.0001
Abdominal pain	4	2077	0.19	36	Fixed	1.12 [0.84, 1.50]	0.44
Constipation	3	2013	0.60	0	Fixed	1.48 [1.09, 1.99]	0.01
**Metabolism/Nutrition Toxicities**							
Decreased appetite	6	2383	0.13	41	Fixed	1.39 [1.18, 1.63]	<0.0001
Decreased weight	5	2118	0.69	0	Fixed	1.45 [1.20, 1.75]	<0.00001
**Hematological Toxicities**							
Platelet count decreased	5	1395	0.70	0	Fixed	1.60 [1.24, 2.06]	0.0002
**Renal /Urinary Toxicities**							
Proteinuria	7	3019	0.11	42	Fixed	2.44 [1.96, 3.04]	<0.00001
**Skin/Subcutaneous Tissue Toxicities**							
Palmar-plantar erythrodysesthesia syndrome	6	2349	0.21	30	Fixed	0.53 [0.46, 0.60]	<0.00001
**General Toxicities**							
Fatigue	7	2949	<0.00001	93	Random	2.13 [0.99, 4.56]	0.05
**Vascular Toxicities**							
Hypertension	9	3158	<0.00001	88	Random	2.43 [1.49, 3.96]	0.0004
**Endocrine Toxicities**							
Hypothyroidism	7	3019	0.02	61	Random	7.09 [3.13, 16.06]	<0.00001

Note: The magnitude of heterogeneity was quantitatively assessed using the I² statistic. When *I^2^* was ≤ 50 %, indicating no statistical heterogeneity among the study results, a fixed-effects model was used. When *I^2^* was > 50 %, indicating the presence of statistical heterogeneity among the study results, a random-effects model was adopted. A p-value > 0.05 suggests the absence of statistical significance, whereas a p-value ≤0.05 indicates the presence of statistical significance.

**Table 3:** Meta-analysis results of adverse event (> 3 grade).

Outcomes	Studies	Sample size	Heterogeneity		Effect model	Meta-analysis results	
			p	*I^2^*		95 % CI	p
**Gastrointestinal Toxicities**							
Nausea	3	2013	0.71	0	Fixed	1.24 [0.41, 3.78]	0.71
Diarrhea	6	2326	0.96	0	Fixed	1.08 [0.67, 1.77]	0.75
Vomiting	2	1908	0.91	0	Fixed	1.16 [0.39, 3.44]	0.79
Abdominal pain	4	2077	0.73	0	Fixed	0.73 [0.39, 1.39]	0.34
Constipation	2	1908	0.70	0	Fixed	4.98 [0.58, 42.54]	0.14
**Metabolism / Nutrition Toxicities**							
Decreased appetite	5	2285	0.88	0	Fixed	4.20 [2.04, 8.63]	<0.0001
Decreased weight	3	2013	0.96	0	Fixed	2.61 [1.52, 4.49]	0.0005
**Hematological Toxicities**							
Platelet count decreased	4	1291	0.37	4	Fixed	1.42 [0.87, 2.32]	0.16
**Renal / Urinary Toxicities**							
Proteinuria	5	2244	0.91	0	Fixed	3.85 [2.06, 7.22]	<0.0001
**Skin / Subcutaneous Tissue Toxicities**							
Palmar-plantar erythrodysesthesia syndrome	4	2349	0.96	0	Fixed	0.29 [0.19, 0.45]	<0.00001
**General Toxicities**							
Fatigue	3	2012	0.87	0	Fixed	1.12 [0.65, 1.93]	0.68
**Vascular Toxicities**							
Hypertension	5	2244	0.64	0	Fixed	1.57 [1.26, 1.96]	<0.0001

Note: The magnitude of heterogeneity was quantitatively assessed using the *I^2^* statistic. When *I^2^* was ≤50 %, indicating no statistical heterogeneity among the study results, a fixed-effects model was used. When *I^2^* was > 50 %, indicating the presence of statistical heterogeneity among the study results, a random-effects model was adopted. A p-value > 0.05 suggests the absence of statistical significance, whereas a p-value ≤0.05 indicates the presence of statistical significance.

#### Metabolism/Nutrition toxicities

The outcome indicators of metabolism/nutrition toxicities included any grade or above and mainly included decreased appetite and weight. The results of the meta-analysis showed that, ① in all levels of metabolism/nutrition toxicities, lenvatinib induced metabolism/nutrition toxicities (RR = 1.41, 95 % CI: 1.25‒1.60, *p* < 0.0001), which was significantly higher than that of the sorafenib group, and the difference was statistically significant. Subgroup analysis showed decreased appetite (RR = 1.39, 95 % CI: 1.18‒1.63, *p* < 0.0001) and decreased weight (RR = 1.45, 95 % CI: 1.20‒1.75, *p* < 0.0001), medium and lenvatinib had higher risk, with statistical difference; ② In tertiary and higher levels of metabolism/nutrition toxicities, lenvatinib caused metabolism/nutrition toxicities (RR = 3.15, 95 % CI: 2.05‒4.85, *p* < 0.00001). The risk was significantly higher than that of the sorafenib group, with statistical significance; Subgroup analysis showed decreased appetite (RR = 4.20, 95 % CI: 2.04‒8.63, *p* < 0.0001) and weight (RR = 2.61, 95 % CI: 1.52‒4.49, *p* = 0.0005) compared with the sorafenib group, and the difference was statistically significant, as shown in Table 2, Table 3.

#### Hematological toxicities

The hematological toxicity outcome index mainly extracted the platelet count decrease data. The results of the meta-analysis showed that at any level of hematological toxicity, lenvatinib caused a decrease in platelet count (RR = 1.60, 95 % CI: 1.24‒2.06, *p* = 0.0002). This risk was significantly higher than that in the sorafenib group, and the difference was statistically significant. In hematological toxicities above grade 3, lenvatinib caused a decrease in platelet count (RR = 1.42, 95 % CI: 0.87‒2.32, *p* = 0.16). The relative risk was similar to that of the sorafenib group, but the difference was not statistically significant (Table 2, Table 3).

#### Renal/Urinary toxicities

The Renal/Urinary toxicity outcome index mainly extracted proteinuria data. The meta-analysis results showed that lenvatinib caused proteinuria of any grade (RR = 2.44, 95 % CI: 1.96‒3.04, *p* < 0.00001), and proteinuria of grade 3 and above (RR = 3.85, 95 % CI: 2.06‒7.22, *p* < 0.0001) relative risks were significantly higher than those in the sorafenib group, with statistical significance, as shown in Table 2, Table 3.

#### Skin/Subcutaneous tissue toxicities

The skin/subcutaneous issue toxicity outcome index mainly extracted the palmar-plantar erythrodysesthesia syndrome data. The results of the meta-analysis showed that lenvatinib-induced palmar-plantar erythrodysesthesia syndrome (RR = 0.53, 95 % CI: 0.46‒0.60, *p* < 0.00001) and grade 3 or higher palmar-plantar erythrodysesthesia syndrome (RR = 0.29, 95 % CI: 0.19‒0.45, *p* < 0.00001) relative risks were significantly lower than those in the sorafenib group, and the differences were statistically significant, as shown in Table 2, Table 3.

#### General toxicities

The fatigue data were mainly extracted from the general toxicity outcome index. The results of the meta-analysis showed that the relative risk of fatigue caused by lenvatinib was slightly higher than that of the sorafenib group (RR = 2.13, 95 % CI: 0.99‒4.56, *p* = 0.05), with statistical significance. The relative risk of fatigue of grade 3 or higher (RR = 1.12, 95 % CI: 0.65‒1.93, *p* = 0.68) was similar to that of the sorafenib group without statistical significance, as shown in Table 2, Table 3.

#### Vascular toxicities

The vascular toxicity outcome index mainly extracted the hypertension data. The results of the meta-analysis showed that lenvatinib-induced hypertension of any grade (RR = 2.43, 95 % CI: 1.49‒3.96, *p* = 0.0004) and higher grade III hypertension (RR = 1.57, 95 % CI: 1.26‒1.96, *p* < 0.0001) relative risks were significantly higher than those in the sorafenib group, and the differences were statistically significant, as shown in Table 2, Table 3.

#### Endocrine toxicities

Endocrine toxicity outcome indices were mainly extracted from the hypothyroidism data. The meta-analysis results showed that the thyroid hormone levels induced by lenvatinib (RR = 7.09, 95 % CI: 3.13‒16.06, *p* < 0.00001) were significantly higher than those in the sorafenib group, and the differences were statistically significant. In both groups, no tertiary hypertension data were extracted (Table 2, Table 3).

### Publication bias test

**Fig. 5:**
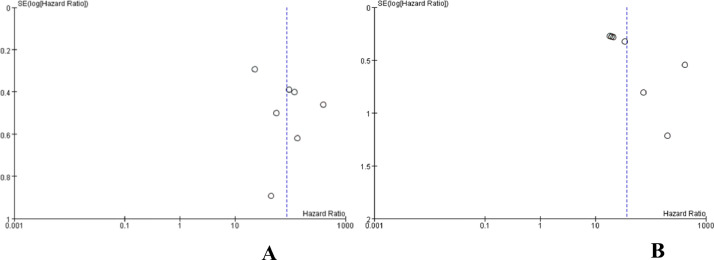
(A) Funnel diagram of PFS. (B) Funnel diagram of OS.

In this study, 10 studies were included, and the funnel plot was used to study publication bias. The funnel plot analysis showed that the PFS and OS results showed that the funnel plot scatter points were asymmetrical, distributed in the middle and upper part of the study, and concentrated toward the middle, indicating that the sample size of this study was large, and the research accuracy was high. At the same time, this suggested that there was a certain publication bias, and the causes of asymmetry in the funnel plot were highly correlated with heterogeneity, as shown in Fig. 5A and B.

## Discussion

Hepatocellular Carcinoma (HCC) is the fourth most prevalent malignant tumor in China and has a profound impact on the quality of life of the Chinese populace.^18^ The absence of pain nerves within the liver obscures the onset of HCC, often resulting in disease progression to an advanced stage at the time of diagnosis. Currently, surgical resection remains the optimal therapeutic approach and is frequently complemented by adjuvant treatments such as radiofrequency ablation and hepatic arterial chemoembolization. However, the efficacy of these interventions is limited, translating into a dismally low five-year survival rate of only 18 %.^19^

Advancements in proteomics research have fostered a deeper understanding of the molecular signaling pathways and microenvironments associated with liver cancer among experts and scholars. Consequently, novel treatment strategies, namely, targeted therapy and immunotherapy, have emerged as focal points in the clinical management of modern advanced HCC. Following the advent of sorafenib, a pioneering targeted drug for first-line clinical treatment of advanced HCC, Tyrosine Kinase Inhibitors (TKIs), a class of small-molecule targeted drugs directed against angiogenesis, have proliferated and flourished. Both first- and second-line drugs primarily function by targeting and inhibiting multiple pathways and variations involved in the genesis and progression of HCC, thereby impeding tumor revascularization and controlling recurrence^20^

Sorafenib is a novel multi-target antitumor drug and small-molecule multi-kinase inhibitor. Comprehensive in vivo and in vitro studies have underscored its capacity to dismantle tumor microvasculature by inhibiting angiogenesis and cellular proliferation, ultimately suppressing tumor growth.^21^ This drug potently inhibits the tyrosine kinase activity of various receptors, including VEGFR-1, −2, −3, PDGFR, FGFR, RET, FLT, and c-Kit. In doing so, it halts tumor angiogenesis, diminishes the nutritional supply to tumor cells, and thereby curtails tumor proliferation and metastasis.^22^^,^^23^ Furthermore, sorafenib inhibits tumor cell proliferation by targeting tyrosine kinase in the upstream receptors of KIT and FLT-3, as well as serine‑threonine kinase in the downstream RAF-MEK-ERK pathway. It exhibits dual inhibitory effects on tumor angiogenesis by targeting upstream VEGFR-2 and PDGFR receptor tyrosine kinases, as well as downstream Raf serine‑threonine kinase, effectively blocking the receptor tyrosine kinase signal transduction pathway and exerting a multifaceted antitumor effect.^24^ Chen et al.^25^ observed a significant reduction in DSA angiography density among patients undergoing sorafenib treatment compared to pretreatment images, affirming sorafenib's ability to inhibit VEGF receptor activity and thereby hinder tumor angiogenesis. In vitro experiments have further demonstrated the efficacy of sorafenib in inhibiting Raf-1 activity at low concentrations, impacting the Ras/Raf/MEK/MAPK signaling cascade, which directly curbs tumor cell proliferation upon inhibition.^26^ Raf kinase plays a pivotal role in regulating extracellular signals and transcription factors, inhibiting apoptosis, and modulating cell differentiation.^27^ MEK serves as a crucial juncture in this signaling pathway, as it activates ERK, a central figure in the cellular signal transduction network. The bispecific recognition and activation mechanism of MEK significantly enhances signal transduction accuracy, thereby preventing erroneous ERK activation.^28^ Liu et al.^29^ also validated that sorafenib can inhibit Raf kinase activity in liver cancer cell lines, subsequently blocking the MEK/ERK signal transduction pathway and decreasing CyclinD1 levels, thereby inhibiting tumor cell proliferation.

Lenvatinib, a multikinase inhibitor, has emerged as another first-line targeted therapy following sorafenib for the treatment of advanced Hepatocellular Carcinoma (HCC). This drug selectively inhibits various proto-oncogenes, including VEGFR, FGFR, PDGFR, RET, and KIT.

In a controlled trial conducted by Kudo et al.,^27^ lenvatinib demonstrated superior efficacy compared with sorafenib in patients with HCC. Specifically, the median Overall Survival (mOS), median Time to Progression (mTTP), and Objective Response Rate (ORR) for lenvatinib were 13.6-months, 8.9-months, and 24 %, respectively, outperforming the sorafenib-treated control group (12.3-months, 3.7-months, and 9 %). Additionally, Choi et al.^28^ found lenvatinib to be more clinically effective than sorafenib, particularly in hepatitis B-related HCC, with comparable incidence rates of adverse reactions (92.34 % vs. 93.09 %). Consequently, lenvatinib is often the preferred option for patients in regions with a high incidence of hepatitis B infection, such as Asia and Africa.

Common adverse reactions associated with lenvatinib include hypertension, anorexia, and fatigue, with a generally higher incidence than that observed with sorafenib.^30^ Although lenvatinib resistance is infrequently reported, it can occur because of mutations such as Neurofibromin-1 (NF1) deletion, which reactivates the PI3K/AKT and MAPK/ERK signaling pathways, or Dual-Specific Phosphatase-9 (DUSP9) loss, which activates the MAPK/ERK signaling pathway.^31^ Furthermore, ETS-1 (E26 Transformation-Specific Sequence-1) upregulation can affect the RAS/MEK/ERK signaling pathway by enhancing VEGFR2 expression.^32^

Both lenvatinib and sorafenib are approved as first-line molecular targeted drugs for advanced HCC and are oral multi-kinase inhibitors. Although their clinical effectiveness as frontline therapies has been established, there is no definitive consensus on which drug is superior.

Recent research^33^ has revealed the immunomodulatory properties of lenvatinib. In immunodeficient mice, lenvatinib exhibited antitumor activity comparable to that of sorafenib, but demonstrated superior efficacy in immunocompetent animals. These findings suggest that combining lenvatinib with immunotherapy may offer new therapeutic options for patients with advanced HCC. Cost-utility analyses have also found lenvatinib to be more cost-effective than sorafenib.^34^ Additionally, lenvatinib has a positive impact on conversion therapy for unresectable HCC, providing patients with significant survival benefits.

The preliminary efficacy, safety, maximum tolerated dose, pharmacokinetics, and pharmacodynamics of lenvatinib in patients with advanced HCC were first reported in Phase I studies in 2015.^35^ Twenty patients were enrolled and stratified based on their Child-Pugh (CP) score into CP-A (9-patients) and CP-B (11-patients). According to the criteria of RECIST-1.1, the overall objective response rate and disease control rate were 15 % (95 % CI: 3.2‒37.9) and 65 % (95 % CI: 40.8‒84.6), respectively. The median progression-free survival was 5.4-months in the CP-A group and 3.6-months in the CP-B group.

This granular risk comparison directly informs the benefit-risk assessment for individual patients, especially those with specific comorbidities. Furthermore, by synthesizing data on recommended doses (12 mg qd for CP-A patients), this review provides a practical reference for clinicians and a foundation for future studies. In 2016, a single-arm, open-label, multicenter Phase II clinical study of lenvatinib was published.^36^ A total of 46 patients participated in the study and received 12 mg qd of lenvatinib over a 28-day treatment cycle. The results revealed a median progression-free survival of 7.4-months, with 17 patients (37 %) achieving partial remission and 19 (41 %) maintaining stable disease. Consequently, the disease control rate was 78 %, and tumor shrinkage was observed in 80 % of the patients. Furthermore, the median overall survival time was 18.7-months, surpassing the outcomes of a multicenter, controlled, non-randomized Phase II study of sorafenib in patients with advanced HCC.

This meta-analysis underscores the effectiveness of lenvatinib in advanced HCC by demonstrating significant improvements in Overall Survival (OS) and Progression-Free Survival (PFS) compared with sorafenib. These findings align with those of a retrospective study involving 41 HCC patients with Portal Vein Tumor Thrombosis (PVTT) of Vp3/4 stage and Child-Pugh class A, which reported enhanced median survival (187-days vs. less, *p* = 0.004) and overall response rate (ORR: 53.8 % vs. 14.3 %, *p* = 0.0193) with lenvatinib monotherapy. Furthermore, lenvatinib outperformed sorafenib in improving Time to Progression (TTP) and ORR among HCC patients with PVTT I-IV, with a median target tumor diameter of 9.8 cm, without increasing the risk of hospitalization or death. Consistent with prior research,^35^ lenvatinib exhibited favorable outcomes despite a higher incidence of adverse reactions than sorafenib. From a biological mechanism perspective, lenvatinib, as an oral multi-target tyrosine kinase receptor inhibitor, selectively inhibits the kinase activity of multiple key receptors, such as Vascular Endothelial Growth Factor (VEGF), thereby blocking the formation of neovascularization in tumors and inhibiting tumor growth and metastasis. This demonstrates its unique advantage as a broad-spectrum tyrosine kinase inhibitor. Although sorafenib also possesses multiple inhibitory effects, lenvatinib has shown superior performance to sorafenib in terms of Progression-Free Survival (PFS) and Overall Survival (OS) in clinical trials. Specifically, in a clinical study targeting patients with advanced primary liver cancer, the average PFS and median OS of the lenvatinib group were significantly longer than those of the sorafenib group. This discovery has made lenvatinib an important treatment option for patients with liver cancer and is also applicable to the treatment of various other types of tumors. The excellent efficacy of lenvatinib not only provides patients with more treatment time and opportunities and improves quality of life and survival rates, but also has relatively controllable adverse reactions, further enhancing patient tolerability and treatment adherence. However, drug selection should be based on the specific condition of the patient and the doctor's advice for careful consideration.

In Phase I, II, and III clinical trials, lenvatinib-induced adverse reactions, common among small-molecule tyrosine kinase inhibitors, include hypertension, Palmar-Plantar Erythrodysesthesia Syndrome (PPES), anorexia, nausea, vomiting, and fatigue.^36^ Most were transient or reversible and manageable through dosage reduction or symptomatic treatment. A comprehensive analysis of tyrosine kinase inhibitor-related adverse reactions by Rimassa et al.revealed a 75 % incidence rate among lenvatinib-treated patients with hypertension (23 %), diarrhea (4 %), anorexia (5 %), weight loss (8 %), fatigue (4 %), PPES (3 %), proteinuria (6 %), elevated blood bilirubin (7 %), and thrombocytopenia (5 %). The less common adverse reactions included nausea, abdominal pain, vomiting, constipation, and dysphonia.^37^ Notably, Hirooka et al.^38^ reported three cases of destructive thyroiditis due to lenvatinib, leading to drug withdrawal in two cases and dosage reduction in one. Sato-Sano et al.^39^ described a case of penetrating skin disease associated with lenvatinib that responded to local corticosteroid therapy, with one lesion persisting two years post-treatment. These rare and potentially severe adverse reactions warrant vigilance in clinical practice.

The present study highlights that lenvatinib poses a significantly higher risk than sorafenib across various categories of gastrointestinal, metabolism/nutritional, hematological, renal/urinary, vascular, and endocrine chemicals. Specifically, lenvatinib significantly elevated the risk of metabolism/nutritional toxicity, renal/urinary toxicity, and vascular toxicity (grade ≥ 3) compared to sorafenib. Conversely, lenvatinib showed a lower incidence of skin/subcutaneous toxicity at any level than sorafenib. In accordance with other studies, the incidence of treatment-related adverse events, including serious and grade ≥ 3 events, was comparable between the lenvatinib and sorafenib groups with similar drug discontinuation rates. Consistent with the safety findings of the REFLECT trial, lenvatinib-treated patients exhibited less palmoplantar erythrodysesthesia and rash, but more hypertension, proteinuria, diarrhea, bilirubin elevation, hypoalbuminemia, and hypothyroidism than sorafenib-treated patients. Notably, the incidence of ascites was significantly higher in the lenvatinib group, suggesting greater hepatotoxicity.^40^

Certain limitations of this study should be acknowledged. First, a meaningful subgroup analysis based on prognostic factors such as tumor stage or liver function grade was precluded by the lack of stratified data reporting in the source publications. The limited number of studies and the small sample sizes within potential subgroups would have resulted in an underpowered analysis with highly uncertain findings. However, the assessment of the baseline characteristics revealed that these key prognostic factors were generally well-balanced between the treatment arms across the included studies, which mitigates the risk of significant confounding of the overall results. The authors therefore maintain that the current pooled effect estimates are robust; definitive conclusions regarding treatment efficacy in specific patient subpopulations await future, adequately powered, and well-designed studies that are committed to the granular reporting of outcomes. Second, the study heterogeneity limits further analysis. Additionally, while potential publication bias did not significantly affect the conclusion, more studies are needed for refinement. Nevertheless, the clinical potential of lenvatinib for HCC treatment is undeniable.

## Conclusion

In conclusion, lenvatinib as a first-line treatment for HCC significantly enhanced PFS and OS. However, the relatively high incidence of certain adverse reactions necessitates close patient monitoring and symptomatic management to enhance the overall safety.

## Abbreviations

HR, Hazard Ratio; RR, Relative Risk; CI, Confidence Interval; MD, Mean Difference; SMD, Standardized Mean Difference; HCC, Hepatocellular Carcinoma.

## Ethics approval and consent to participate

Ethics approval and consent to participate are not applicable.

## Consent for publication

Consent for publication is not applicable.

## Availability of data and materials

The raw data supporting the conclusions of this article will be made available by the authors, without undue reservation.

## Authors' contributions

W. B and J. Z, conceived the study and wrote the paper; Z.W, XH. D aided in the collection of data. The authors read and approved the final manuscript.

## Funding

There is no funding support for this project.

## Declaration of competing interest

The authors declare no conflicts of interest.

## Appendix Supplementary materials

Image, application 1
